# First-Principles Investigation of Excited-State Lattice Dynamics and Mechanical Properties in Diamond

**DOI:** 10.3390/mi16060668

**Published:** 2025-05-31

**Authors:** Ying Tian, Fangfang Meng, Duanzheng Wu, Dong Yang, Xiaoma Tao, Zian Li, Jau Tang, Xiang Sun, Junheng Pan

**Affiliations:** 1 School of Physical Science and Technology, Guangxi University, Nanning 530004, Chinazianli@gxu.edu.cn (Z.L.); 2 Beijing National Laboratory for Condensed Matter Physics, Institute of Physics, Chinese Academy of Sciences, Beijing 100190, China; 3 The Institute of Technological Sciences, Wuhan University, Wuhan 430072, China; jautang@whu.edu.cn

**Keywords:** diamond, electron occupation, excited-state, structural evolution, mechanical property

## Abstract

The study of the excited-state properties of diamond is crucial for understanding its electronic structure and surface physicochemical properties, providing theoretical support for its applications in optoelectronic devices, quantum technologies, and catalysis. This research employs Density Functional Theory (DFT) with the fixed electron occupation method to simulate the electron excitation. Using the Generalized Gradient Approximation (GGA) within DFT, we systematically investigated the excited-state characteristics of diamond by simulating the transfer of a fraction of electrons from the Highest Occupied Crystal Orbital (HOCO) to the Lowest Unoccupied Crystal Orbital (LUCO). Theoretical calculations indicate that with increasing electron excitation levels, the diamond crystal structure transitions from cubic to tetragonal, accompanied by a gradual decrease in the bandgap. Mechanical property analysis reveals that both Young’s modulus and shear modulus decrease with increasing excitation rate, while the bulk modulus remains nearly constant. These findings indicate a significant impact of electronic excitation on the mechanical stability of diamond. Phonon dispersion curves exhibit reduced degeneracy in high-frequency optical branches and a marked decrease in crystal symmetry upon excitation. This study not only advances the understanding of diamond’s excited-state properties but also offers valuable theoretical insights into its structural evolution and performance tuning under such extreme conditions.

## 1. Introduction

As a representative wide-bandgap semiconductor, diamond is indispensable in fields such as mechanical machining [1], electronic devices [2,3], and quantum computing [4] owing to its exceptional hardness [5], outstanding thermal conductivity [6], high carrier mobility [7], wide bandgap, and superior chemical stability [8]. However, advancing the understanding and enabling effective modulation of these properties necessitates a systematic investigation of diamond’s microscopic structure and dynamics under excited states to establish the intrinsic correlation between atomic vibrations and macroscopic physical characteristics. In recent years, rapid advancements in fields such as optoelectronic device miniaturization, breakthroughs in quantum computing technologies, and catalytic science have driven a growing demand for research on the structural evolution and mechanical properties of diamond under excited-state conditions.

Experimental characterization of the atomic-scale structure and dynamic evolution of diamond in the excited state remains a significant challenge. Traditional techniques such as Raman spectroscopy [9], X-ray diffraction [10], and neutron scattering [11] are primarily used to investigate the structural information of diamond in its ground state. Although time-resolved spectroscopic techniques can capture the relaxation processes of excited-state diamond, they generally lack the spatial resolution required to resolve atomic-scale structural evolution [12,13,14,15]. Recently developed techniques such as ultrafast electron diffraction (UED) [16] and ultrafast X-ray diffraction (UXRD) [17] provide promising avenues for directly probing the dynamic evolution of diamond’s lattice structure in the excited state. These methods hold the potential to reveal the electron–phonon coupling and structural dynamics of diamond following excitation. However, such advanced experimental techniques impose extremely stringent requirements on instrumentation performance and experimental conditions. This is particularly true for diamond, whose strong covalent bonds and high crystal symmetry make it exceptionally challenging to accurately resolve subtle structural changes and mechanical responses, such as variations in elastic constants, under excitation. These experimental limitations highlight the critical need for the development of fast, reliable, and efficient theoretical approaches to model excited-state behavior.

Accurate calculations of the excited-state properties of diamond, such as excitation energy, optical transitions, and structural evolution, are crucial for advancing its applications in optoelectronics and quantum information science. However, simulating these excited-state behaviors remains a significant challenge, as the excited state is inherently a many-body problem that involves complex electron correlation effects, which are beyond the scope of ground-state density functional theory (DFT). While high-precision computational methods, such as quantum Monte Carlo (QMC) [18,19] and many-body perturbation theory (GW-BSE) [20,21], can effectively describe excited-state processes, their high computational cost severely limits their applicability in simulating the excited-state properties of materials. Therefore, finding a balance between accuracy and efficiency, as well as developing more efficient computational methods, has become a key research direction in this field.

Currently, various DFT-based excited-state computational methods have been employed to investigate diamond and its defect states. Among these, time-dependent density functional theory (TD-DFT), an extension of ground-state DFT, has been widely used to study the single-photon and two-photon absorption properties [22], singlet optical transitions [23], optical bandgap $E_{g}$ predictions [24], and optical absorption spectra [25] of nitrogen-vacancy defects in diamond. However, TD-DFT still has limitations when dealing with complex excited states, such as charge-transfer excitations, multiple excitations, and strongly correlated systems. The Delta Self-Consistent Field (ΔSCF) method simulates specific excitation processes by explicitly controlling the occupation of electronic states beyond the ground state. It has been employed to investigate the excited-state energy levels of the ${\mathrm{NV}}^{-}$ color center in diamond, successfully reproducing the energy level ordering of the triplet and singlet states [26]. This method avoids the complexity of TD-DFT and many-body perturbation theory calculations, but it exhibits convergence difficulties. Constrained Density Functional Theory (CDFT) simulates specific excited states by imposing additional constraints on the electron or spin density. It is particularly effective for describing localized excitations, such as charge-transfer processes or specific spin configurations. CDFT has been used to describe excitations between the singlet states of the ${\mathrm{NV}}^{-}$ color center in diamond [27]. However, the accuracy of this approach heavily depends on the physical justification of the imposed constraints. In the first-principles computational package VASP.6.3.0 (Vienna *Ab initio* Simulation Package), electronic excitations can be simulated by manually fixing the occupation numbers of Kohn–Sham orbitals through specific input parameters. This method is conceptually similar to ΔSCF or orbital-constrained CDFT approach. It has been used to study the electronic structure of singlet states in hexagonal boron nitride (h-BN) [28], the energy of defect-related electronic states [29], and the origin of defect-related AB emission lines in the photoluminescence (PL) spectrum of 4H-SiC [30]. Therefore, the fixed electron occupation method for simulating excited-state properties, with a computational cost similar to ground-state DFT and high computational efficiency, shows strong potential for predicting excited-state properties in materials such as diamond.

In this study, the fixed electron occupation method based on DFT is employed to simulate the electronic excitation process, with all calculations performed using the VASP code. The transfer of electrons from the Highest Occupied Crystal Orbital (HOCO) to the Lowest Unoccupied Crystal Orbital (LUCO) is precisely controlled to systematically investigate the lattice structure evolution of diamond in the excited state. To quantitatively characterize the dynamic response of diamond’s mechanical properties in the excited state, the Voigt–Reuss–Hill method [31,32,33] is introduced to establish a quantitative correlation model between the bulk modulus (*B*), shear modulus (*G*), Young’s modulus (*E*), and excitation intensity. Compared with traditional research methods, this approach constructs the structure–property relationships for the electronic excitation rate, crystal structure, and mechanical properties of diamond, elucidating the mechanism by which electronic excitation modulates the mechanical properties of diamond. This provides a theoretical foundation for the design of diamond-based devices under extreme conditions.

## 2. Methods

All calculations were performed using the VASP code [34]. The exchange-correlation functional was primarily selected as the Perdew–Burke–Ernzerhof (PBE) functional within the generalized gradient approximation (GGA) [35]. For mechanical property calculations, the local density approximation (LDA) functional was also used for comparison to assess the impact of different functionals on the results. The structural model consisted of a cubic unit cell containing 8 carbon atoms, with the initial lattice constant set to 3.57 Å×3.57 Å×3.57 Å, consistent with experimental values [36]. To ensure computational accuracy, the mesh cutoff energy value was set to 520 eV. The Brillouin zone was sampled using the Monkhorst–Pack scheme [37], with a k-point mesh of 3 × 3 × 3. The total energy convergence criterion for the self-consistent field (SCF) calculations was set to $1\times 10^{-8}$ eV/cell. During the geometric structure optimization process, the conjugate gradient algorithm was used to fully relax the atomic positions and lattice parameters, until the force on each atom was smaller than $5\times 10^{-4}$ eV/Å, at which point convergence was considered achieved. To eliminate temperature effects, all calculations were performed under 0 K conditions, and van der Waals interaction corrections were introduced to improve the accuracy of the calculations.

The excited-state simulations were performed by precisely controlling the number of electron transfers between the HOCO and LUCO. The electron excitation rate (η) is defined as:

(1) $$\eta =\frac{N_{\mathrm{ex}}}{N_{\mathrm{total}}}$$

where $N_{\mathrm{ex}}$ is the number of excited electrons, and $N_{\mathrm{total}}$ is the total number of electrons in the system. By tuning the value of $N_{\mathrm{ex}}$, the electron excitation rate η was precisely controlled within the range of 0% to 6.25%, enabling the investigation of the structural and mechanical properties of diamond under varying excitation conditions.

Elastic constants are key parameters that characterize the mechanical properties of materials, directly related to the crystal’s mechanical stability, stress–strain response, and intrinsic hardness. For cubic diamond (space group $Fd\bar{3}m$), the elastic constants $C_{ij}$ have three independent components: $C_{11}$, $C_{12}$, and $C_{44}$. Upon electron excitation, the diamond structure transforms into a tetragonal phase (space group $I4_{1}/amd$), resulting in six independent elastic constants: $C_{11}$, $C_{12}$, $C_{13}$, $C_{33}$, $C_{44}$, and $C_{66}$.

The elastic constants of crystals with different space group symmetries must satisfy specific mechanical stability conditions. For cubic diamond, the mechanical stability conditions are:

(2) $$C_{11}>0,\;C_{44}>0,\;C_{11}>\left|C_{12}\right|,\;\left(C_{11}+2C_{12}\right)>0$$

When diamond transitions from the cubic phase to the tetragonal phase, the mechanical stability conditions are as follows:

(3) $$\begin{array}{cc} \; & C_{11}>0,\;C_{33}>0,\;C_{44}>0,\;C_{66}>0,\\ \; & \left(C_{11}-C_{12}\right)>0,\;\left(C_{11}+C_{33}-2C_{13}\right)>0,\\ \; & \;\left[2\left(C_{11}+C_{12}\right)+C_{33}+4C_{13}\right]>0, \end{array}$$

In this study, the Voigt–Reuss–Hill (VRH) averaging scheme [31,32,33] was employed to calculate the elastic moduli. The specific calculation formulas are as follows:

(4) $$B=\frac{1}{2}\left(B_{V}+B_{R}\right),\;G=\frac{1}{2}\left(G_{V}+G_{R}\right)$$

In these expressions, $B_{V}$, $B_{R}$, $G_{V}$, and $G_{R}$ represent the bulk and shear moduli estimated using the Voigt and Reuss approximations [38], respectively. The Young’s modulus (*E*) and Poisson’s ratio (ν) can be derived from the bulk and shear moduli as follows:

(5) $$E=\frac{9GB}{3B+G},\;\nu =\frac{3B-2G}{2(3B+G)}$$

## 3. Results

**Figure 1:**
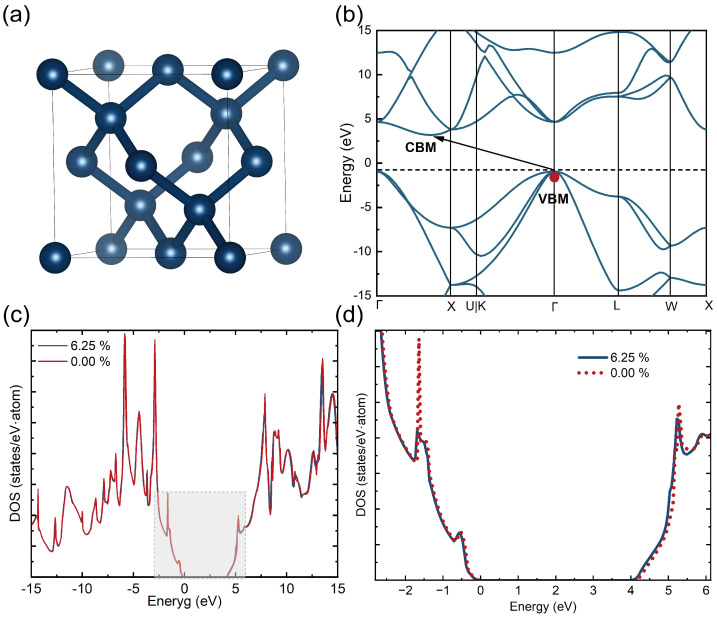
Electronic structure of diamond. (**a**) Crystal structure of diamond. (**b**) Electronic band structure of pristine diamond calculated using the PBE functional, with the excitation mechanism indicated. (**c**) Total density of states (DOS) for the ground state and the excited state (6.25%). (**d**) Enlarged view of the density of states near the valence band maximum (VBM) and conduction band minimum (CBM). The results show that the bandgap decreases with increasing electronic excitation, accompanied by a reduction in electron occupation in the valence band and an increase in electron population in the conduction band.

The face-centered cubic (FCC) unit cell of diamond was adopted as the initial structure (Figure 1a). The calculated band structure (Figure 1b) shows a characteristic indirect bandgap, with arrows indicating the electron transfer from the valence band maximum (VBM) to the conduction band minimum (CBM). A structural phase transition from the initial FCC phase to a tetragonal phase was induced by adjusting the electron occupation via the fixed electron occupation method, followed by full structural optimization. Further analysis reveals that as the electron excitation rate η increases, the lattice expands equally in the a and b directions ($\Delta a/a_{0}=\Delta b/b_{0}$), while exhibiting significant contraction along the c direction ($\Delta c/c_{0}<0$). The total energy increases monotonically from −72.678 eV to −72.338 eV, without any significant energy fluctuations throughout the process. This anisotropic lattice deformation is strongly correlated with the changes in bond lengths induced by electron excitation.

Figure 1c compares the density of states (DOS) of diamond in its ground state (η=0.00%, red curve) and excited state (η=6.25%, blue curve). Figure 1d shows the orbital DOS near the Fermi level (−2~6 eV). The comparison reveals a narrowed bandgap in the excited state, and the electron distribution near the Fermi level changes significantly. To quantitatively characterize this change, the DOS was numerically integrated over specific energy intervals for both the ground and excited states to evaluate the electron occupancy near the Fermi level. The results indicate that in the −2~0 eV range, the integral value for the ground state is 0.832, while in the excited state it is 0.755. In the 4~6 eV range, the ground state integral is 0.805, while the excited state increases to 0.849. These results suggest that following the change in electron occupation, electron occupancy decreases below the Fermi level and increases above it. Moreover, the excited electrons are primarily filled in the conduction band minimum. This feature is consistent with the band structure characteristics shown in Figure 1b, further validating the electron transition mechanism from the VBM to CBM.

**Figure 2:**
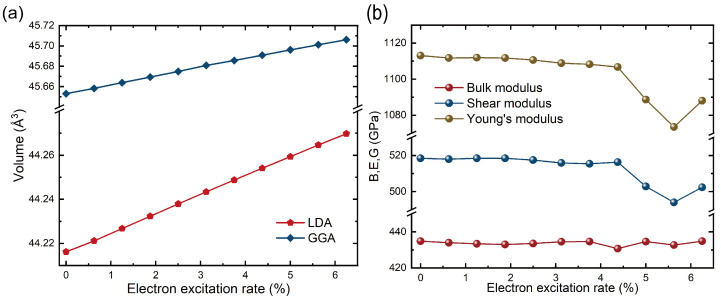
(**a**) Relationship between unit cell volume and electronic excitation calculated using LDA and GGA functionals. (**b**) Variation trends of the bulk modulus (B), shear modulus (G), and Young’s modulus (E) of diamond with increasing electronic excitation, aiming to reveal the influence of electronic excitation on its elastic properties.

Figure 2a illustrates the variation of the unit cell volume with the electron excitation rate η. A comparison between the GGA-PBE (blue squares) and LDA (red hexagons) results reveals a clear volume expansion trend in both cases. At an excitation rate of 6.25%, the GGA functional yields a volume increase of 1.16‰, while the LDA functional predicts a slightly larger expansion of 1.21‰, indicating that LDA is more sensitive to excitation-induced structural changes. Furthermore, we analyzed the impact of changes in electron occupation states on the mechanical properties of diamond. Based on the calculated elastic constants at different excitation states, the bulk modulus (*B*) and shear modulus (*G*) were extracted to quantify the corresponding mechanical responses.

Table 1 presents the calculated elastic constants of diamond in both the ground and excited states. The ground state elastic constants are calculated using the GGA-PBE and LDA functionals, and all results satisfy the mechanical stability criteria [38]. The GGA-PBE results exhibit better agreement with experimental values [39], with the maximum deviation remaining below 3.2%. Based on this analysis, all subsequent calculations for the excited states were carried out using the GGA-PBE functional.

Table 2 summarizes the elastic moduli of diamond in both the ground and excited states, which are critical for evaluating its suitability in engineering applications. According to the Pugh ratio (B/G), materials with a B/G value greater than 1.75 are considered ductile, while those with a value below 1.75 are classified as brittle. The results obtained via the Voigt–Reuss–Hill averaging method show that the B/G ratios for all excited states fall within the range of 0.84~0.88, indicating that diamond maintains its intrinsic brittleness under electronic excitation. Additionally, the Poisson’s ratio (ν) is used to evaluate the material’s ductility and brittleness. When ν>1/3, the material is considered ductile, whereas ν<1/3 indicates a brittle material. Across all excitation states investigated, the Poisson’s ratio remains below 0.1 (ranging from 0.072 to 0.086), further confirming the brittle nature of diamond under the conditions studied.

Figure 2b shows the qualitative relationship between the bulk modulus (*B*), shear modulus (*G*), Young’s modulus (*E*), and effective electron excitation rate. The results reveal that both the shear and Young’s moduli exhibit an overall inverse correlation with increasing excitation rate, whereas the bulk modulus remains relatively constant, with fluctuations within 0.96%. In the low excitation regime (η<4.375%), both the shear and Young’s modulus decrease gradually with increasing excitation rate, indicating a slight weakening of diamond’s rigidity. However, when the excitation electron rate exceeds 4.375%, both moduli experience a sharp drop, indicating a sudden change in the material’s mechanical properties. As the excitation rate increases further to 5.625%, both moduli show a recovery trend.

To investigate the underlying mechanism behind this sudden change, we calculated the relative growth rate of the C–C bond length compared to the ground state under different excitation states. The results show that at excitation rates of 4.375%, 5.000%, 5.625%, and 6.250%, the growth rates of the C–C bond length are $1.623\times 10^{-3}$, $-1.203\times 10^{-3}$, $-1.347\times 10^{-3}$, and $3.204\times 10^{-3}$, respectively. This indicates that around an excitation rate of approximately 5.000%, the bond length growth rate significantly decreases, and a recovery trend is observed at 5.625%. Notably, when the excitation rate exceeds 4.375%, the C–C bond length change rate exhibits non-monotonic fluctuations ($\Delta d/d_{0}$ decreases from +0.162% to −0.135%). This anomalous change in bond length response may lead to a reduced ability of the diamond lattice to coordinate with the applied stress field.

**Figure 3:**
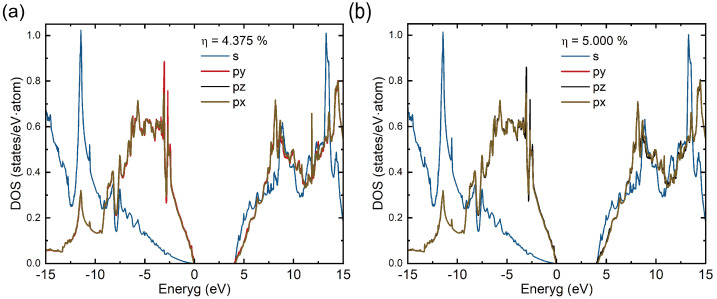
Projected density of states (PDOS) at 4.375% (**a**) and 5.000% (**b**) excitation rates. A comparative analysis reveals a directional shift in bonding, transitioning from the initial in-plane (xy) orientation to an out-of-plane (axial) configuration.

The PDOS analysis (Figure 3) reveals that within the excitation rate η range of 4.375% to 5.000%, there is a significant anisotropic redistribution of electrons in the p-orbitals of carbon atoms: the electron occupation in the px/py orbitals (in-plane direction) decreases, while that in the pz orbitals (axial direction) increases. This directional transfer of orbital occupation alters the spatial distribution of the electron cloud, which in turn induces localized structural changes. This is a key factor responsible for the abrupt change in the elastic modulus.

**Figure 4:**
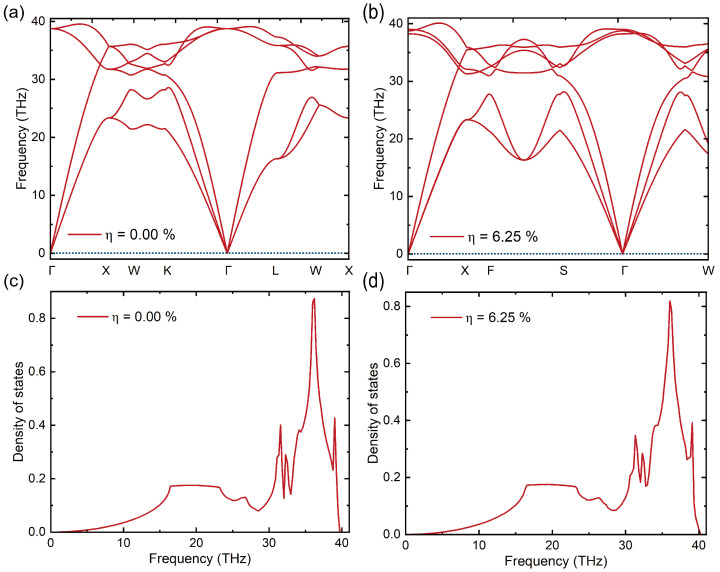
Phonon band structures (**a**,**b**) and phonon density of states (**c**,**d**) for the ground state and the excited state (6.25%). As shown in panels (**a**,**b**), the excited state exhibits significant changes in the high-frequency optical phonon modes, including the emergence of transverse mode splitting, indicating a reduction in crystal symmetry induced by electronic excitation. The PDOS plots further support this observation, showing subtle changes in the high-frequency region around 30 THz in the excited state compared to the ground state, confirming that the phonon properties are influenced by electronic excitation.

A 2×2×2 supercell model was constructed to calculate the phonon band structure and corresponding phonon density of states of diamond in both the ground state (η = 0.00%) and the excited state (η = 6.25%), along high-symmetry directions in the first Brillouin zone, as shown in Figure 4. As illustrated in Figure 4a,b, no imaginary frequencies appear throughout the Brillouin zone, confirming the dynamical stability of the excited-state structure despite the altered electronic configuration. Compared with the ground state, the degeneracy of the optical branches in the excited state is significantly reduced. A distinct splitting of the transverse optical modes is observed along the Γ–W direction (Figure 4b), indicating a reduction in crystal symmetry induced by electronic excitation. Further analysis of the phonon density of states reveals that the dominant vibrational modes are concentrated in the high-frequency region (30–35 THz).

## 4. Discussion

The fixed electron occupation method employed in this study constructs non-ground-state electronic excitations by predefining the occupation numbers of Kohn–Sham orbitals, followed by self-consistent field (SCF) iterations until convergence is achieved. This approach reveals a phase transition in diamond from the cubic phase ($Fd\bar{3}m$) to the tetragonal phase ($I4_{1}/amd$) under electronic excitation—a phenomenon not yet reported experimentally in diamond. Under high-intensity femtosecond laser excitation, the potential energy surface (PES) between atoms in diamond undergoes significant changes [40]. Molecular dynamics (MD) simulations show that femtosecond laser excitation can suppress the local minimum in the PES corresponding to the diamond structure, thereby driving an ultrafast (100 fs) non-equilibrium structural phase transition, such as the transformation from diamond (sp³) to graphite (sp²) [41]. This represents a key mechanism of non-thermal phase transitions induced by electronic excitation. The structural transformation of diamond simulated in this study corresponds to the early stage of such a phase transition. Under thermal annealing conditions, a temperature-dependent mechanism for symmetry reduction in nanodiamond structures has been observed. Below 900 °C, surface reconstruction of nanodiamond leads to graphitization, while above 900 °C, the diamond core itself undergoes graphitization, marked by a change in carbon hybridization from sp³ to sp² [42,43]. While our study investigates an excitation-induced phase transition, it is interesting to note that structural transformations are a common response mechanism in strong covalent carbon systems under various stimuli. For instance, studies on complex diamond-like phases under mechanical tension have shown that the critical limit before failure is often not a fracture but a phase transformation into a short-ordered lattice [44]. This suggests that bond rearrangement is a preferred pathway over catastrophic bond breaking in these robust materials.

It is noteworthy that group-IV elements such as silicon and germanium exhibit similar phase transitions under high pressure. Experimental studies have shown that under hydrostatic conditions, silicon transforms from the cubic phase ($Fd\bar{3}m$) to the tetragonal β-Sn structure ($I4_{1}/amd$) at approximately 12 GPa, accompanied by a semiconductor-to-metal transition and a 20% volume collapse [45,46,47]. Under laser shock compression, this transition pressure can be reduced to 5.4(5) GPa [48]. Similarly, germanium transitions from the cubic phase (Ge I) to the tetragonal phase (Ge II) in the 9–12.5 GPa range [49]. First-principles calculations further confirm that the transition pressures for Si and Ge under hydrostatic conditions are 11.4 GPa and 9.5 GPa, respectively, while under uniaxial compression, they are reduced to 3.9 GPa and 2.5 GPa [50]. Although the tetragonal phase of diamond has not been experimentally observed, DFT calculations have predicted the existence of several tetragonal carbon allotropes [51,52], offering theoretical insight into the design of novel carbon-based materials.

The calculated bulk modulus, shear modulus, and Young’s modulus of diamond in the ground state are presented in Table 2. These values are consistent with experimental measurements, which report a bulk modulus of 442 GPa, a shear modulus of 535 GPa, and a Young’s modulus of 1050 GPa [39,53,54,55]. As the electronic excitation rate increases, both the shear modulus and Young’s modulus exhibit a decreasing trend, consistent with the well-established observation that elastic constants typically decrease under tensile stress [56]. Furthermore, the analysis of elastic constants indicates a degradation of the mechanical properties of diamond under excited states. In this study, we evaluated the elastic properties of polycrystalline diamond and therefore did not consider the directionality of the elastic moduli.

The main advantage of the fixed electron occupation method lies in its high computational efficiency. Compared to TD-DFT or many-body perturbation theories, it offers significantly lower computational cost, making it well-suited for studying the structural and phonon properties of materials under excited-state conditions. By controlling the external iterative procedure, this method enables full orbital relaxation while maintaining spatial and spin symmetries. Moreover, it allows for the determination of the properties of wavefunctions converged by VASP and facilitates finding the lowest-energy wavefunctions and energies under any given spatial and spin symmetry. It can also converge to higher-energy states with specific spatial and spin symmetry [29]. However, it is important to note that this method is time-independent and therefore may not accurately capture non-adiabatic dynamics or electron-hole correlations. The interpretation of results obtained from this approach must be carried out with caution, as convergence to excited states is only conditionally valid. Since it relies on standard DFT functionals, it often fails to accurately describe electron-phonon coupling and tends to significantly underestimate the electron-phonon coupling (EPC) strength [57]. In some cases, changes in orbital energies may cause the electron density to collapse back to the ground state, making convergence difficult [58]. Therefore, future investigations could consider incorporating more advanced computational approaches, such as TD-DFT or the GW approximation, along with electron–phonon coupling (EPC), to more precisely describe the excited-state properties of diamond. Machine learning has been successfully applied to analyze the mechanical and vibrational properties of carbon nanotubes [59]. In the field of diamond-based materials, machine learning methods are expected to be used in studies related to their structure and mechanical properties. Additionally, this study mainly focuses on the response of diamond under relatively low excitation rates, while the effects of higher excitation levels remain unexplored. In practical applications, however, diamond may be subjected to high-energy radiation or intense light exposure, leading to significantly higher excitation rates. Future research could thus explore the structural stability, electron relaxation dynamics, and potential phase transitions of diamond under such extreme excitation conditions.

## 5. Conclusions

Understanding the behavior of diamond under far-from-equilibrium conditions, particularly in highly excited electronic states, is of great significance for the advancement of high-power electronic devices, quantum technologies, and advanced materials processing. In this study, we employed a fixed electron occupation approach within the framework of DFT to systematically investigate the structural stability, electronic structure, mechanical response, and lattice vibrational properties of diamond under strong electronic excitation. Our results reveal a series of excitation-induced phenomena, including a phase transition from a cubic to a tetragonal structure, bandgap narrowing, elastic modulus softening, and a reduction in the degeneracy of high-frequency optical phonon branches. These findings not only demonstrate the tunability of diamond’s fundamental physical properties through electron excitation but also provide theoretical insights into its behavior under extreme conditions, thereby expanding its potential applications in specialized technological scenarios.

## Figures and Tables

**Table 1:** The elastic constants $C_{ij}$ (in GPa) for diamond in its ground state and various excited states, along with a comparison to experimental results.

Excitation Rate (%)	$C_{11}$	$C_{12}$	$C_{13}$	$C_{33}$	$C_{44}$	$C_{66}$
$0.000^{LDA}$	1027.83	123.49			542.75	
$0.000^{GGA}$	1050.68	126.97			560.07	
0.625	1049.96	125.21	126.51	1049.45	559.57	558.71
1.250	1049.75	126.82	124.37	1049.76	559.69	559.69
1.875	1049.58	127.27	123.48	1049.58	559.67	559.67
2.500	1049.40	122.59	127.67	1047.24	559.78	556.67
3.125	1046.52	128.10	128.09	1049.20	555.92	559.70
3.750	1045.84	128.46	128.46	1049.13	555.21	559.85
4.375	1045.29	128.72	119.78	1049.11	554.34	559.98
5.000	1044.10	129.02	129.02	1049.17	553.10	500.17
5.625	1049.12	117.62	129.26	1044.08	500.30	552.99
6.250	1043.33	129.69	129.68	1049.05	552.36	500.38
Experiment [39]	1079	124			578	

**Table 2:** Bulk modulus *B* (GPa), Young’s modulus *E* (GPa), shear modulus *G* (GPa), and Poisson’s ratio ν of diamond in the ground state and at various excited states.

Excite Rate (%)	$B_{V}$	$B_{R}$	$G_{V}$	$G_{R}$	*B*	*G*	*E*	ν
$0.000^{LDA}$	424.94	424.937	506.52	502.486	424.937	504.502	1084.369	0.075
$0.000^{GGA}$	434.88	434.887	520.79	516.165	434.877	518.475	1113.076	0.073
0.625	433.98	433.981	520.31	515.764	433.981	518.038	1111.752	0.073
1.250	433.37	433.375	520.73	516.184	433.375	518.455	1111.950	0.072
1.875	433.02	433.021	520.77	516.239	433.022	518.504	1111.776	0.072
2.500	433.54	433.544	519.79	515.230	433.544	517.508	1110.620	0.073
3.125	434.53	434.533	518.17	513.592	434.533	515.882	1108.838	0.075
3.750	434.62	434.618	517.75	513.164	434.618	515.456	1108.244	0.075
4.375	430.69	430.692	518.49	514.198	430.693	516.346	1106.752	0.072
5.000	434.61	434.608	504.63	501.079	434.609	502.853	1088.683	0.083
5.625	432.73	432.730	495.13	492.876	432.732	494.003	1073.506	0.086
6.250	434.87	434.865	504.13	500.587	434.866	502.359	1088.088	0.083

## Data Availability

All data that support the findings of this study are included within the article.

## Abbreviations

The following abbreviations are used in this manuscript:

DFT	Density Functional Theory
HOCO	Highest Occupied Crystal Orbital
LUCO	Lowest Unoccupied Crystal Orbital
PES	Potential Energy Surface
TD-DFT	Time-Dependent Density Functional Theory
CDFT	Constrained Density Functional Theory
EPC	Electron-Phonon Coupling
Δ SCF	Delta Self-Consistent Field
